# Supplementary figures and images for: Single-cell transcriptomic atlas of enteroendocrine cells along the murine gastrointestinal tract
Source: PLoS One. 2024 Oct 8;19(10):e0308942. doi: 10.1371/journal.pone.0308942 (PMC11460673; doi:10.1371/journal.pone.0308942)

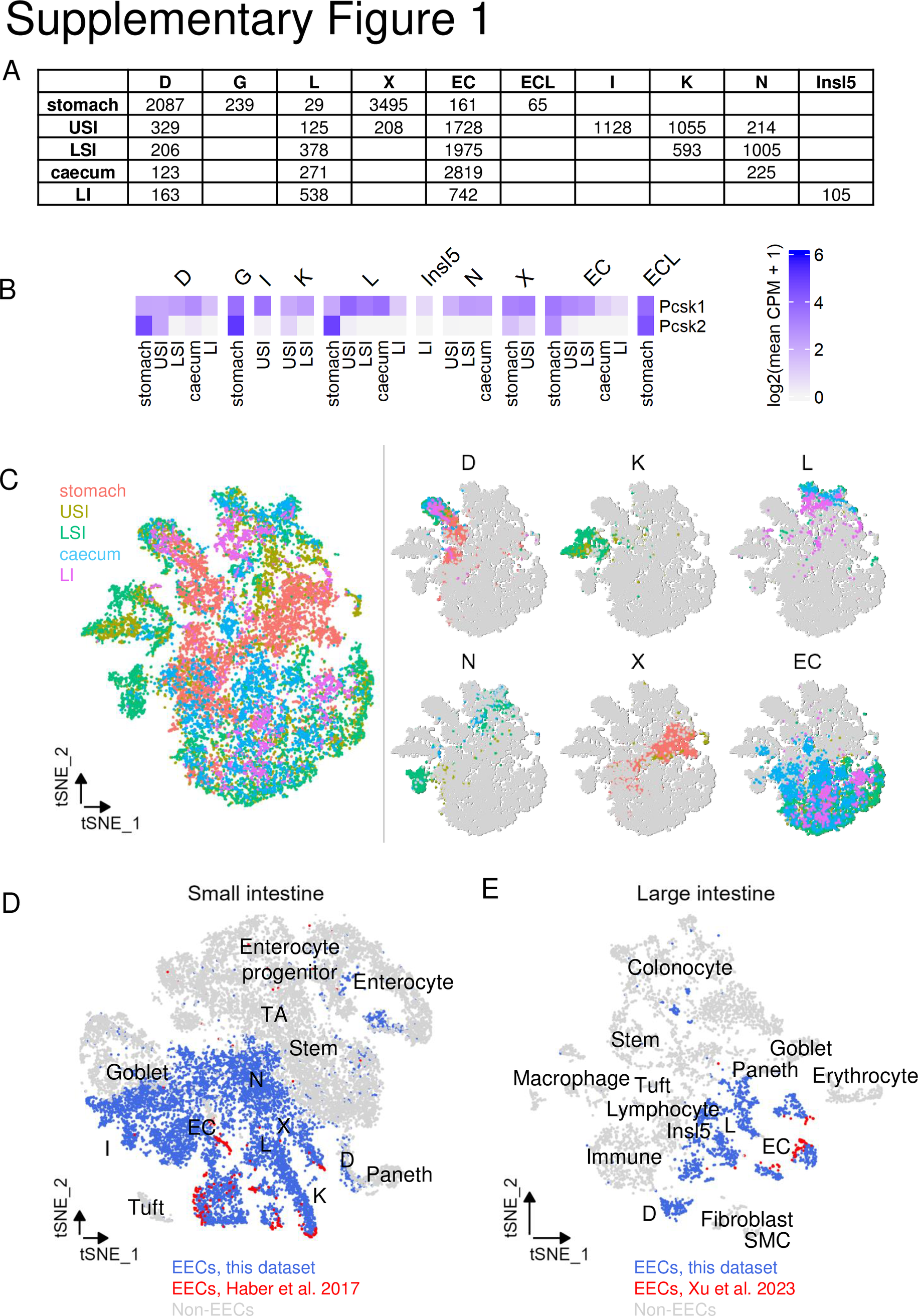

Supplement: S1 Fig — (A) Numbers of cells in each cluster from Fig 1A. (B) Expression of Pcsk1 and Pcsk2 in each EEC clusters per GI region. Expression is log2(mean counts per million + 1). (C) tSNE plot for integrated dataset of all EECs from all GI regions. Left: Samples labelled by GI region. Right: Samples labelled by GI region for D, K, L, N, X, and ECs only. (D,E) Data from the small and large intestine were separately integrated with previously published single cell RNA sequencing datasets, including (D) mouse small intestine epithelial cells (7) and (E) wild-type mouse colon cells [18]. tSNE maps show overlap in EECs from the published (red) and our dataset (blue), amongst non-EECs (grey) from each dataset. (TIF) [file pone.0308942.s001.tif]

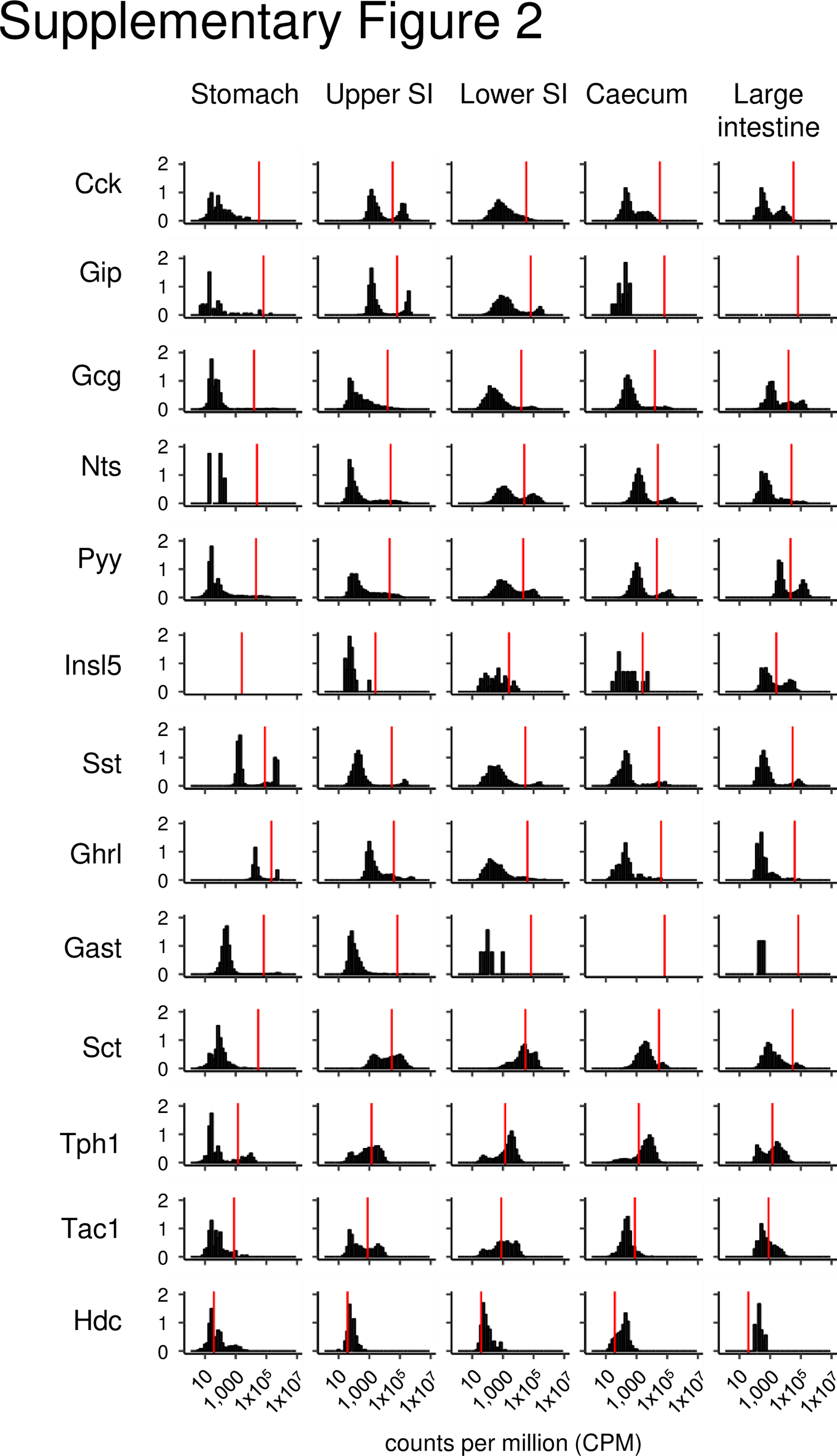

Supplement: S2 Fig — Histogram of raw counts per EEC marker (left to right) per GI region (top to bottom). Huang threshold labelled by red vertical line. (TIF) [file pone.0308942.s002.tif]

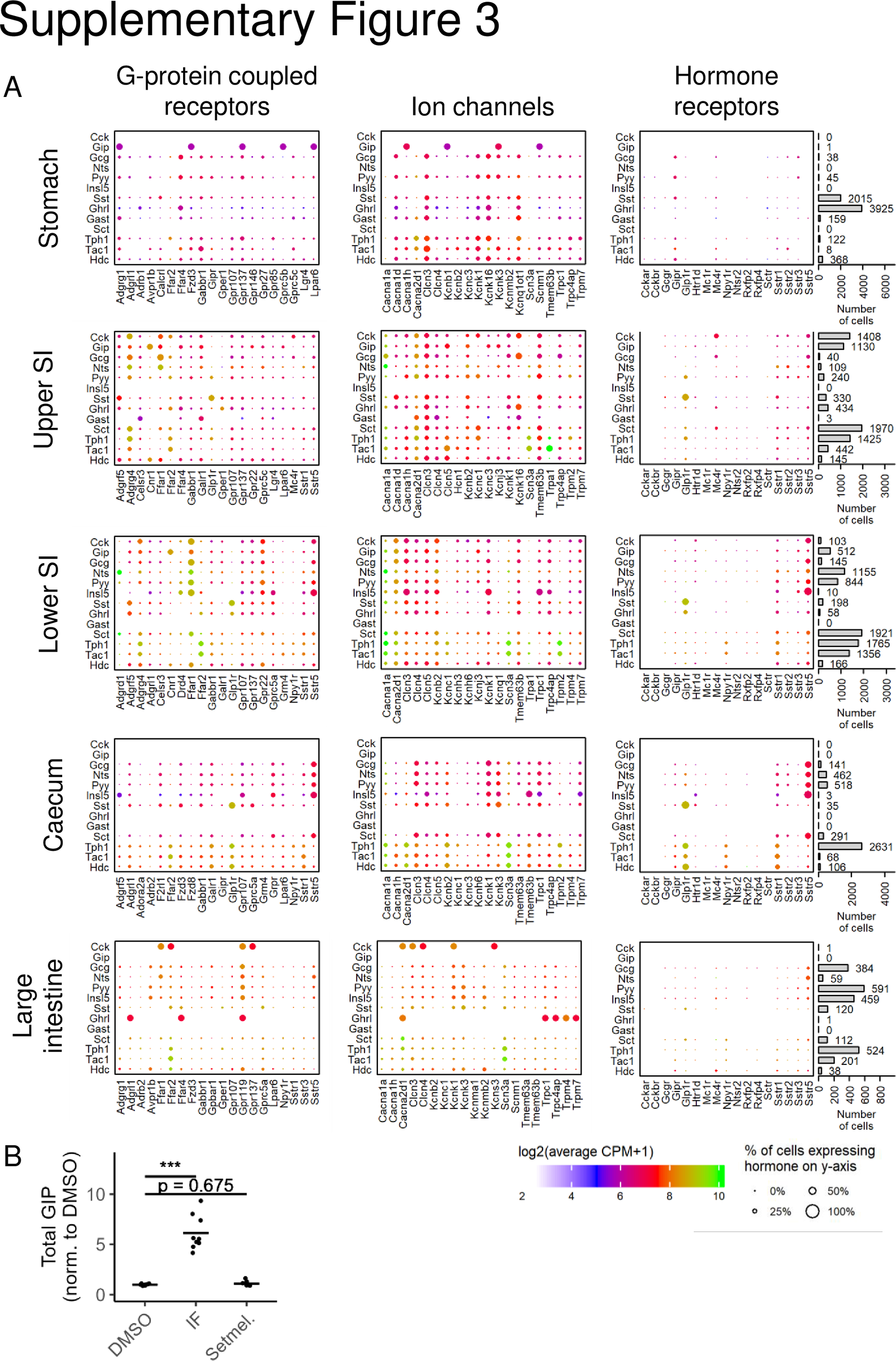

Supplement: S3 Fig — Dot plots representing co-expression of hormones (or Tph1) and (A) GPCRs, (B) ion channels, and (C) enteric hormone receptors, per GI region. Dot size represents the percentage of cells expressing the hormone on the y-axis, dot colour the expression of the gene on the x-axis in log2(mean counts per million + 1). Bars on the right represent the number of cells expressing the hormone on the y-axis. GPCR and ion channel genes in (A) and (B) are selected as the top 20 most-expressed differentially-expressed (padj < 0.05) genes of that type, per GI region. (B) Total GIP secretion, measured by ELISA, from mouse USI primary tissue after 2 hr stimulation with setmelanotide (10 μM), with DMSO (2%), and IBMX (10 μM)/forskolin (10 μM)(FI) as negative and positive controls, respectively. Concentrations in test supernatants are shown normalised to mean concentrations in DMSO controls, per experiment. Horizontal lines represent the mean (n = 3 experiments, 3 samples per experiment). Statistical analysis by two-way ANOVA, and Tukey’s HSD post-hoc test (*** p.adj < 10−3). (TIF) [file pone.0308942.s003.tif]
