# Supplementary material for: Single-cell transcriptomic atlas of enteroendocrine cells along the murine gastrointestinal tract
Source: PLoS One. 2024 Oct 8;19(10):e0308942. doi: 10.1371/journal.pone.0308942 (PMC11460673; doi:10.1371/journal.pone.0308942)
Supplement: Attachment — Submitted filename: Responses to reviewers.docx [file pone.0308942.s004.docx]

Responses to reviewers’ comments

Reviewer #1:

Enteroendocrine cells (EECs) are rare hormone-producing cells in the intestinal epithelium. Distinct subtypes of EECs produce unique combinations of hormones, which regulate metabolism, bowel movement and appetite. Due to their scarcity, defining EEC heterogeneity at the transcriptome level has been challenging. In this study, the authors utilize a genetically encoded EEC reporter to generate a scRNAseq atlas comprising ~20K EECs from the proximal to distal mouse gastrointestinal tract. They describe regional variations in EEC subtype abundance, and showcase several GPCRs with a biased expression in specific EEC subtypes. As functional validation, they demonstrate that a specific GPCR agonist induces EEC activation in vitro. Overall, this study provides a comprehensive resource for the EEC field. Due to the existence of multiple single cell RNA atlases in the mouse gut that cover many EECs, this advance is however rather incremental. Please find below my point-by-point suggestions.

- In figure 3: It is unclear to what extent reclustering based on GPCR expression allowed the authors to discover differences in GPCR distribution across EEC subtypes. It seems likely that differential expression on the initial clustering would have revealed the same.

Response: We agree with the reviewer that there are different ways in which the GPCR distribution can be analysed across different EEC populations. In S3 Fig we took a traditional approach and analysed individual GPCR expression across different EEC populations defined by their expression of a hormone of interest. As expected, this revealed differential GPCR across EEC populations. The aim of the experiment in figure 3 was to test the reverse hypothesis, that the EEC populations differ by how they signal. We believe this is an interesting alternative approach to compare cell populations, and the finding of differential Mc4r expression in CCK-cells was one of the key outcomes.

- Figure 4: The authors should include measurements of other hormones in response to Mc4r agonists, to stress the Cck-specific signaling.

Response: We thank the reviewer for making this suggestion and have performed additional experiments to test the hypothesis that Mc4r agonism would not affect secretion of GIP. As predicted, setmelanotide increased secretion of CCK but not of GIP from primary mouse upper small intestinal epithelial cultures. The additional CCK results have been added to the original data, increasing the n-number, and the GIP results are shown in a new subfigure (S3 Fig). As these were upper small intestinal cultures, GLP-1 and PYY levels were too low to quantify, so we restricted the new analysis to CCK and GIP.

- The authors succinctly write in lines 418/419 that the identification of Mc4r in I cells is interesting, as previous work showed its role in GLP-1 release. Reference 24 does indeed show this/L cell expression, but also evidenced I cell expression using a CCK-reporter mouse. The authors should therefore mention Mc4r was previously found to be enriched in I cells.

Response: We thank the reviewer for noticing that we had not referred to the CCK-cell data in this reference. This is now included, and the text reads:

“The identification of Mc4r in I-cells was interesting as this receptor has previously been implicated in GLP-1 release from the distal gut, and has been identified but not functionally characterised in EECs purified from Cck-Cre mice (25, 26)”

- It appears that in figure 4B the legend erroneously indicates that the dot size represents the percentage of cells expressing a certain hormone, instead of a GPCR.

Response: Thank you, this is now corrected.

- Is there any bias in isolating certain EEC subtypes using the NeuroD1 tracer? All EEC subsets seem to be present, but a statement/reference on this would be helpful. Stomach endocrine cell distribution appears different than reported (should be more biased for EC/ECL). In addition: could the authors comment on the distribution of ECL cells across GI tract regions. It is present in equal numbers across the GI tract, while exclusive presence in the stomach is expected. This is also stated in line 294 (the authors similarly state this is absolute for G cells, which again is found in other regions).

Response: As also questioned by reviewer 2, we have now systematically examined whether the NeuroD1-Cre reporter labelled EECs in an unbiassed manner. As shown in the new S1 Fig (D, E), we combined our new scRNAseq data with published scRNAseq data from the epithelium of the upper SI (Haber et al 2017) and large intestine (Xu et al 2023). Our EEC populations showed good overlap with the (relatively small) EEC populations in these unbiassed datasets, and we did not identify any EEC populations in the unbiassed data that had not been detected using the NeuroD1 marker. The revised version contains additional methods, and the following text in the results:

“We combined the data from our USI and LSI NeuroD1-labelled EECs with published unbiassed datasets (7, 18). EECs from the current study showed good overlap with EEC populations in the unbiassed datasets (S1 Fig), and no additional EEC populations were evident in the full epithelial data that had not been picked up using the NeuroD1-Cre approach.”

We thank the reviewer for noticing that the Huang thresholding method (shown in S2 Fig as the method used to define positive cells in Fig 2), calculated that there are more ECL cells than expected in regions beyond the stomach. This is because the method looks at the range of expression of the marker gene across all the cells in the region, and calls those with the highest expression as “positive” even if the expression level is very low. Use of a thresholding method is necessary because transcripts for hormones that are highly expressed in a tissue are detectable in the majority of negative cells, likely because high abundance RNA released from a few burst cells can be picked up in all droplets during the 10X analysis. We prefer not to tweak the Huang thresholding programme manually to generate an expected outcome, so have instead added extra colour coded circles in figure 2 to show average expression of the marker gene in its positive cell population in each region. This shows up as a series of circles across the diagonal, with the colour varying by region e.g. Hdc is now seen to be more highly expressed in the Hdc-positive cells in the stomach than in Hdc-positive cells in the colon/rectum (where it is co-expressed at low levels in L-cells). We hope this provides some additional clarity, and have added the following text to explain the data:

“Use of the Huang thresholding method identified similar numbers of EECs as the clustering method for most cell types, but over-estimated ECL cell numbers in regions beyond the stomach where low *Hdc* expression in other EEC types was called positive by the algorithm (Fig 2 A). “

We have also now presented the cell numbers generated from the clustering in fig1A, as an alternative estimate of cell cluster sizes in different regions of the gut (S1 Fig). This again reveals that the Huang thresholding method has worked well for most cell types, but has broken down for ECL cells in regions where both cell abundance and Hdc expression are low.

- I could not find any statement on the average number of reads/genes detected per cell. This would be important, including a comparison to previous murine gut sequencing studies. As much as the number of cells sequenced, the depth per cell is key to identify lowly expressed genes such as GPCRs.

Response: Apologies for the omission of this important piece of information. The following text has now been added to the methods:

“Of the filtered data, the median (min – max) number of reads per cell were 35,679 (2,140 – 389,084) in the stomach, 15,440 (718 – 261,381) in the USI, 6,462 (650 – 216,128) in the LSI, 5,664 (1,648 – 104,978) in the caecum, and 4,438 (1,402 – 40,537) in the LI dataset. The median (min – max) number of genes per cell were 3,276 (1,002 – 9,330) in the stomach, 3,663 (504 – 9,350) in the USI, 2,487 (501 – 9,367) in the LSI, 2,043 (1,001 – 8,308) in the caecum, and 1,943 (1,001 – 5,935) in the LI dataset.”

Reviewer #2:

This manuscript from an eminent laboratory on enteroendocrine cell (EEC) biology compares mouse stomach, proximal and distal small intestine, caecum, and colonic EEC transcriptomes at single-cell resolution. Knowing that EEC physiology is tied to G protein-coupled receptor (GPCR) signaling, the authors highlight differences and overlaps in GPCR mRNA expression across EECs and GI regions. They show that a range of nutrients trigger Ca++ flux in ileal L cells differentiated in vitro.

Several scRNA-seq profiles of EECs have been reported, including some by this group, but this is the first to consider the full length of the mouse GI tract together (colonic EEC data are taken from a previous study that is cited). The work is performed to the high standards of the senior authors, who are recognized experts in the field. The quality of the data seems high and the story is presented clearly. Although certain conclusions, such as that most EECs express more than one bioactive peptide, have been reported previously, these aggregate data from >20,000 cells will make a useful contribution to the literature. Demonstration of cholecystokinin (CCK) release in response to multiple stimulants (Fig. 3B) adds one physiologic dimension to the study.

My only question pertains to the second physiologic dimension: Ca++ flux that various agonists trigger in Venus-labeled L cells isolated from differentiated ileal organoids (Fig. 4C-E). The range of values shown on the graph (Fig. 4D) raising questions about what is real signal and what is background. The implication of the dashed line on the graph is that any R/R0 value >1.0 is signal and therefore legitimate to represent in the Venn diagram (Fig. 4E) as a “reactive” cell. However, the example trace in Fig. 4C suggests that many of the values clustered near 1.0 may represent background noise. Only some of those cells may merit inclusion within the Venn representation. The authors should represent the data with clear and conservative definitions of real signals or establish that even small deviations over 1.0 are real. Data from a larger number of cells may also add clarity and rigor.

Response: We thank the reviewer for noticing that we had not included a description of how we defined a cell as a “responder”. The cut-off for defining responders was set at a relatively conservative increase by 10% in the fura-2 ratio during addition of the test compound. Based on extensive previous experience, this cut-off is more likely to generate false negatives than false positives. This has been added to the methods, which now reads:

“Cells exhibiting increases in the fura2 fluorescence ratio of >10% during perfusion of the test agent were counted as responders – a definition that is more likely to generate false negatives than false positives.”

Reviewer #3:

The study entitled “Single-cell transcriptomic atlas of enteroendocrine cells along the murine gastrointestinal tract” by Smith et al. explores enteroendocrine cells (EECs) diversity and expression along the gastrointestinal tract by single-cell RNA-sequencing of GFP-labeled sorted EECs from different tissues and locations along the tract (from the stomach to rectum). The authors identified ten main EEC clusters within the different tissues with similar transcriptional profiles, showing multiple expressions of a few gut hormones in specific subsets. The authors investigated GPCRs of the different EEC types and showed that GPCR expression correlates with the gene expression of the cells. The authors propose to utilize this approach to test other specific factors within the cells, such as transcription factors, to investigate the diverse EEC population and properties.

My only comment is the disadvantage of the EEC isolation method that relies on a GFP reporter under the specific TF associated with most EECs, NeuroD1. Using this method allows the enrichment of EECs from EpCAM+ cells, which are about 2-4% of total EpCAM, but could miss EEC subsets that are NeuroD1 negative. It would be interesting to compare the data obtained from this study to an unbiased analysis of EECs extracted from the total epithelial cells of one tissue.

Response: We thank the reviewer for this comment, and have now integrated our data with the single cell data from upper small intestine (Haber 2017) and large intestine (Xu 2023). The merged clustering analysis is included in a new S1 Fig D,E. The analyses showed good overlap between the EECs from the different studies, but did not identify any EEC populations in the unbiassed datasets that were not picked up using NeuroD1-Cre as a reporter. We conclude that our analysis did successfully identify all the different EEC populations derived from the gut epithelium in the small intestine, and have added this to the methods and results. The new text in the results is:

“We combined the data from our USI and LSI NeuroD1-labelled EECs with published unbiassed datasets (7, 18). EECs from the current study showed good overlap with EEC populations in the unbiassed datasets (S1 Fig), and no additional EEC populations were evident in the full epithelial data that had not been picked up using the NeuroD1-Cre approach.”

In conclusion, the authors performed elegant experiments to characterize the EEC subsets of different gastrointestinal compartments and test for hormones and GPCRs associated with them.

This manuscript will surely provide valuable data and guidelines for further research in EEC, food intake, and metabolic disorders in general.

Response: Thank you for the positive comments
