# Supplementary material for: Single-cell transcriptomic atlas of enteroendocrine cells along the murine gastrointestinal tract
Source: PLoS One. 2024 Oct 8;19(10):e0308942. doi: 10.1371/journal.pone.0308942 (PMC11460673; doi:10.1371/journal.pone.0308942)
Supplement: Attachment — Submitted filename: Responses to reviewers 2.docx [file pone.0308942.s005.docx]

Reviewer #1:

A remaining note on the cell clustering in Figure 2:
The authors comment that the Huang thresholding calls cell types based on transcript levels are relative to cell in that particular GI tract region. It may be better to combine all regions for the tresholding and then separately present the data? To me it is still somewhat confusing to present these high HDC+ numbers in the distal gut. This may also resolve some other 'stretched' annotations (e.g., now it appears there are relatively equal number of Gcg+ cells in the proximal and distal gut, INSL5 in the proximal gut..). More GIP than GCG in the distal gut? This is perhaps more a point of preference of how to present this, and I appreciate the addition of average gene expression as a clarification.

Response: We thank the reviewer for this question, which triggered us to look yet again at the Huang thresholding method. We tried several ways of combining the data, and have now made 2 adjustments to the method: (1) we have combined data from all regions, as the reviewer suggested, with the exception of *Ghrl* and *Sst* in the stomach which were so highly expressed that they resulted in background levels in other stomach EECs that were higher than in *Ghrl* and *Sst*-expressing cells in the small intestine – this was a major reason we had originally treated each region independently; (2) we have used CPM instead of raw counts when combining the data. The new calculations still identify Hdc-expressing cells in the distal gut, but these are not a distinct cell type, rather there is some Hdc expression in other EECs, particularly enterochromaffin cells and L-cells. We have changed the description of this result to make it clear that this is not a distinct ECL population. The new text reads:

“Low levels of *Hdc* expression were unexpectedly identified in some EECs in the distal intestine, predominantly colocalised in EC cells in the caecum and with L-cell hormones in the large intestine.”

Reviewer #2:

I was the most positive of the 3 referees, with significant concern only about the Fura2A ratios represented in Fig. 4C-E. The authors’ response is that a 10% increase in the ratio is “relatively conservative” and that “based on extensive previous experience,” that “cut-off is more likely to generate false negatives than false positives.” This response and insertion of a sentence to that effect in the Methods are less than satisfactory. Unless I don’t understand the tracing (Fig. 4C) or how values are graphed in Fig. 4D, the distribution of values within any group seems to vary by more than 10% and the responses to Glucose or Tryptophan look very different in Fig. 4C. I don’t challenge the authors’ conservatism, nor do I need to stand in the way of publication, but other readers may share my concern (confusion?) and deserve more clarity about the assay and its signal/noise parameters. I otherwise like this paper and believe it makes a useful contribution to the field.

Response: There is no perfect way of distinguishing a responding cell from a non-responder, since some responses can be subtle and background noise in the traces varies from cell to cell. Attempting to address the reviewer’s concern, we have taken a different approach in this revision, taking 10 readings of the fura2 ratio from just before addition of the test reagent, and 10 centred around the maximum response during perfusion with the test compound, for each cell. We have then applied a t-test between these pairs of readings and called a cell as a responder if there was a significant increase (with p<0.01). I’m not sure this is particularly superior to the 10% method, although it does call some additional cells as responders. I don’t think the exact numbers in each set of the Venn diagram are critical, as the major question was whether the cells are capable of responding to more than one stimulus type. Overall the conclusion of this section is unchanged by the method of analysis: that EECs are multi-responsive to different types of nutrient signal.
